# Recent Progress on Rapid Lateral Flow Assay-Based Early Diagnosis of COVID-19

**DOI:** 10.3389/fbioe.2022.866368

**Published:** 2022-05-03

**Authors:** Ying Zhang, Yujuan Chai, Zulu Hu, Zhourui Xu, Meirong Li, Xin Chen, Chengbin Yang, Jia Liu

**Affiliations:** ^1^ Central Laboratory, Longgang District People’s Hospital of Shenzhen and The Second Affiliated Hospital of the Chinese University of Hong Kong, Shenzhen, China; ^2^ Guangdong Key Laboratory for Biomedical Measurements and Ultrasound Imaging, School of Biomedical Engineering, Health Science Center, Shenzhen University, Shenzhen, China

**Keywords:** lateral flow assay, nucleic acid, antigen, antibody, the point of care, COVID-19, SARS-CoV-2

## Abstract

The outbreak of the coronavirus disease 2019 (COVID-19) has resulted in enormous losses worldwide. Through effective control measures and vaccination, prevention and curbing have proven significantly effective; however, the disease has still not been eliminated. Therefore, it is necessary to develop a simple, convenient, and rapid detection strategy for controlling disease recurrence and transmission. Taking advantage of their low-cost and simple operation, point-of-care test (POCT) kits for COVID-19 based on the lateral flow assay (LFA) chemistry have become one of the most convenient and widely used screening tools for pathogens in hospitals and at home. In this review, we introduce essential features of the severe acute respiratory syndrome coronavirus 2 (SARS-CoV-2) virus, compare existing detection methods, and focus on the principles, merits and limitations of the LFAs based on viral nucleic acids, antigens, and corresponding antibodies. A systematic comparison was realized through summarization and analyses, providing a comprehensive demonstration of the LFA technology and insights into preventing and curbing the COVID-19 pandemic.

## Graphical Abstract

Viral RNA‐, antibody‐, antigen-Based LFAs are used for large-scale screening of COVID‐19 at home, school, and under various non-laboratory scenarios. 
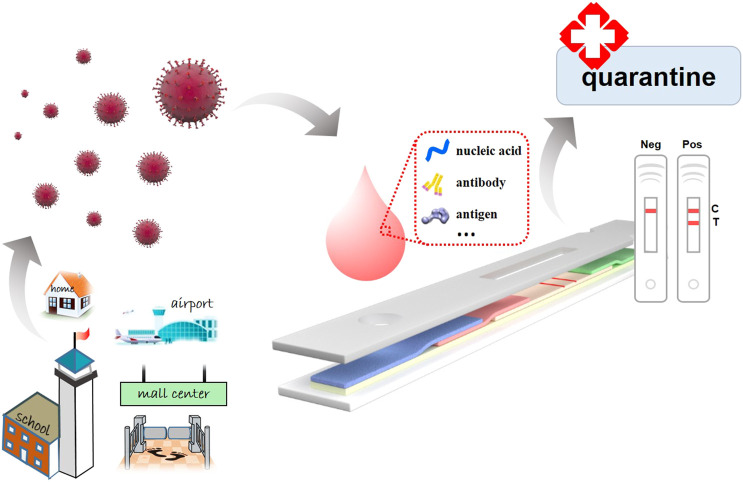

## Introduction

Since the emergence of severe acute respiratory syndrome coronavirus 2 (SARS-CoV-2) in December 2019, this lethal pathogen has developed into a pandemic, causing over 6 million deaths as of April 2022 owing to its high human-to-human transmissibility mediated by airborne droplets (Worldometer, 2022). Studies have shown that the clinical manifestations of SARS-CoV-2 infection are diverse, including asymptomatic infections (only positive nucleic acid test, no clinical symptoms), acute respiratory responses (with respiratory symptoms but no lung imaging changes) and varying degrees of pneumonia (with respiratory symptoms and lung imaging changes) (Lai et al., 2020; Tang et al., 2021). Fever, cough, fatigue, sore throat, and dyspnea are the most common symptoms. Severe cases can rapidly progress to acute respiratory distress syndrome (Rodriguez-Morales et al., 2020; Hu B. et al., 2021). The coronavirus disease 2019 (COVID-19) pandemic has exerted unprecedented pressure on individuals, families, medical systems, and the social economy. One of the most effective methods to control the pandemic is to develop rapid detection techniques to screen and diagnose infected individuals, including asymptomatic carriers, as soon as possible during the incubation period (Mei et al., 2020).

Since the outbreak, researchers worldwide have developed numerous rapid and sensitive diagnostic kits that can be divided into two categories: genetic material-based molecular diagnosis and antigen/antibody-based immunoassays. Nucleic acid quantification methods include quantitative reverse transcription-polymerase chain reaction (qRT-PCR), loop-mediated isothermal amplification (LAMP)-based assays, clustered regularly interspaced short palindromic repeats (CRISPR)-associated protein (Cas) system, and RNA sequencing. qRT-PCR is regarded as the gold standard for COVID-19 detection because of its high sensitivity, reliability, and throughput (Jing et al., 2021). Two steps are essential for the detection of SARS-CoV-2 *via* qRT-PCR: reverse transcription of RNA into complementary DNA (cDNA) and amplification of cDNA by the PCR using conserved primers and fluorescent probes (Nolan et al., 2006; Bustin and Nolan, 2020). Although the qRT-PCR technique is mature and accurate, it is time consuming, expensive in terms of the equipment and reagents, and requires trained technicians for complex sample preparation and testing procedures. These defects restrict the application of the platform to the central laboratory and occasionally lead to inaccurate results owing to insufficient materials or sample transportation problems, thus making the method unsuitable for the rapid, low-cost, and accurate detection of COVID-19 (Li Y. et al., 2020; Liu R. et al., 2020; Kim Y. J. et al., 2020). Similar to qRT-PCR, RT-LAMP is based on a set of four primers and the strand displacement of active reverse transcriptase. It can produce billions of template DNA under isothermal conditions in less than 1 h (Nguyen et al., 2020; Yang et al., 2020). LAMP-based assays have been used for the early detection of COVID-19 owing to their short reaction time, high sensitivity, good stability, and simplified sample preparation steps, such as nucleic acid extraction. Recently, methods for SARS-CoV-2 nucleic acid determination based on the CRISPR system combined with LFAs have been reported. The CRISPR-Cas system was originally known for its powerful function in gene editing (Jinek et al., 2012; Cong et al., 2013). The novel applications of COVID-19 *in vitro* diagnosis are discussed in this review.

Immune-based assays mainly include enzyme-linked immunosorbent assays (ELISA), chemiluminescent immunoassays (CLIA), and lateral flow immunochromatography assays (LFIA), among which ELISA is the classical immunoassay used to detect pathogens (Konstantinou, 2017; Noh et al., 2019). Indirect assays (Adams et al., 2020; Liu W. B. et al., 2020; Xiang et al., 2020), sandwich assays (Lv et al., 2019), and competitive binding assays (Xiang et al., 2020) are common types of ELISA assay. The main advantages of ELISA for COVID-19 diagnosis are high sensitivity and the ability to detect multiple samples in one test run. However, ELISA can only be carried out in the laboratory because the protocol involves multiple steps, has a long turnaround time, and requires both skilled personnel and specific instrumentation. Compared to ELISA, CLIA is frequently performed owing to its automation (Soleimani et al., 2021), high specificity, low interference, short incubation time, and wide dynamic range (Cinquanta et al., 2017). However, the application of CLIA for rapid and large-scale screening of COVID-19 is hampered by the high cost of machinery and maintenance, inflexible operation, and strict environmental requirements for reagent transportation and storage. Based on the principles of molecular detection and immunological testing, a series of new methods for the detection of COVID-19 have been developed by combining electrochemistry, nanomaterials, artificial intelligence, mass spectrometry, and other technologies. They have been summarized in several reviews, including those focused on molecular diagnosis (Zhu H. et al., 2020; Esbin et al., 2020; Feng et al., 2020; Mattioli et al., 2020; Rahimi et al., 2021; Yuce et al., 2021), antibody detection (Wang J. J. et al., 2021; Ejazi et al., 2021), nanotechnology (Mahapatra and Chandra, 2020; Qin et al., 2020; Chintagunta et al., 2021; Kailasa et al., 2021) and others (Carter et al., 2020; Ji et al., 2020; Pokhrel et al., 2020; Udugama et al., 2020; Yuan et al., 2020; Chen et al., 2022; Szunerits et al., 2022). Although these methods are sensitive, their operation is sophisticated, the reagents including fluorophore and enzyme are vulnerable, and they are only restricted to laboratory applications and cannot be widely promoted. The LFA technique for the early diagnosis has great potential for preventing, monitoring, and controlling COVID-19, especially in the post-pandemic era. However, only a few comprehensive reviews have focused on LFA (Habli et al., 2021; Hsiao et al., 2021; Hsieh et al., 2021; Jia et al., 2021; Sadeghi et al., 2021; Yadav et al., 2021; Zhou et al., 2021). In this work, LFA that could be used for large-scale screening of COVID-19 at home, school, and under various non-laboratory scenarios owing to its simplicity, convenience, rapidity, and cost-efficiency is discussed. The principles, advantages and disadvantages of these methods will be discussed in detail to provide guidance and suggestions for the prevention and transmission control of the COVID-19 pandemic in the post-pandemic era from the prospective of a diagnostic strategy. Before summarizing the LFA methods, the structural characteristics and infectivity of SARS-CoV-2 should be emphasized to better understand the molecular mechanisms of the diagnostic approaches.

## Structural Characteristics and Infectivity of SARS-CoV-2

**FIGURE 1:**
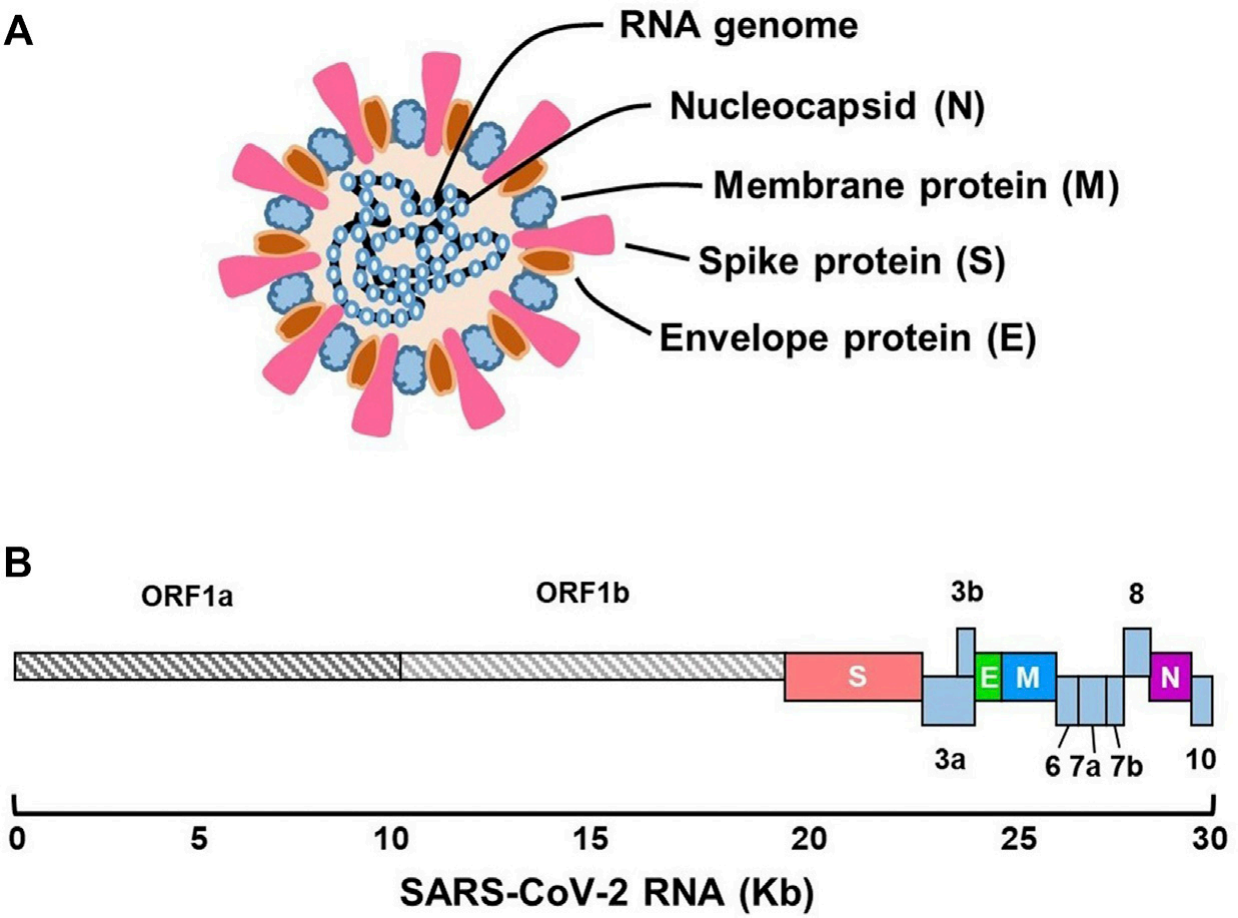
Schematic presentation of the genomic structure of SARS-CoV-2. **(A)** Virus particle and its components. **(B)** Genome organization of different functional regions.

SARS-CoV-2 is a single-stranded positive RNA virus (+ssRNA) with a genome size of 30 kb, belonging to the Coronaviridae family and the beta *Coronavirus* (Zhu N. et al., 2020). The genomic sequence of SARS-CoV-2 was first confirmed by Wu and coworkers (Wu et al., 2020) at the National Center for Biotechnology Information (accession no. MN908947.3). Sequence analysis identified a genome of 29,903 nucleotides encoding 9,860 amino acids. The genome includes 5-′ untranslated region (5-UTR), open reading frames1a/b (ORF1a/b), and structural protein genes-3′-UTR (Figure 1). The coding regions of nonstructural proteins are mainly located in ORF1a and ORF1b. These two fragments occupy approximately two-thirds of the viral genome, encoding 16 nonstructural proteins (nsps). The remaining one-third of the genome encodes structural proteins, including spike (S), membrane (M), envelope (E), and nucleocapsid (N) proteins, as well as auxiliary proteins (Cui et al., 2019; Chen Y. et al., 2020). The S protein is a large trimetric transmembrane glycoprotein that forms a particular corolla structure on the viral surface and contains two subunits: S1 and S2 (Ou et al., 2020). The S1 subunit consists of a signal peptide folded into a receptor-binding domain (RBD) and an N-terminal domain (NTD). The RBD is a crucial component of viral infection because it can directly bind to specific receptors on the surface of host cells (Andersen et al., 2020; Yan R. H. et al., 2020; Lan et al., 2020). The S2 subunit contains a fusion peptide (FP), heptapeptide repeats 1 and 2 (HP1 and HP2), a transmembrane domain, and an intracellular domain, promoting the fusion of the viral and cell membranes. SARS-CoV-S1 and SARS-CoV-2-S1 share approximately 66% amino acid identity, whereas the identity of S2 between them is as high as 90%. Therefore, the structure of the S2 subunit is more conserved, and the antibody against the S1 subunit is more specific (Okba et al., 2020). Another structural protein, the N protein, can interact with viral RNA to form a viral nucleocapsid in a beads-on-a-string-type conformation. This protein plays an important role in the protection, replication, and synthesis of viral RNA (Rosales-Mendoza et al., 2020). The amino acid sequences of the two structural proteins are relatively conserved, which determines the infectivity and structural function of the virus, and they are chosen as the target antigen in many rapid diagnostic tests, especially in LFA. This is discussed in detail in the following section. The E protein is a component of the viral envelope that participates in viral assembly, release, and pathogenicity. It contains transmembrane alpha-helix and hydrophobic domains and acts as an ion channel in the pentamer structure (Verdia-Baguena et al., 2012). Similarly, the M protein contains a conserved region and three transmembrane domains. It is an integral part of the viral envelope and participates in the assembly and release of viruses. In the following paragraph, the process of pathogen infection of the host is briefly reviewed to illustrate the function of each viral structural protein.

**FIGURE 2:**
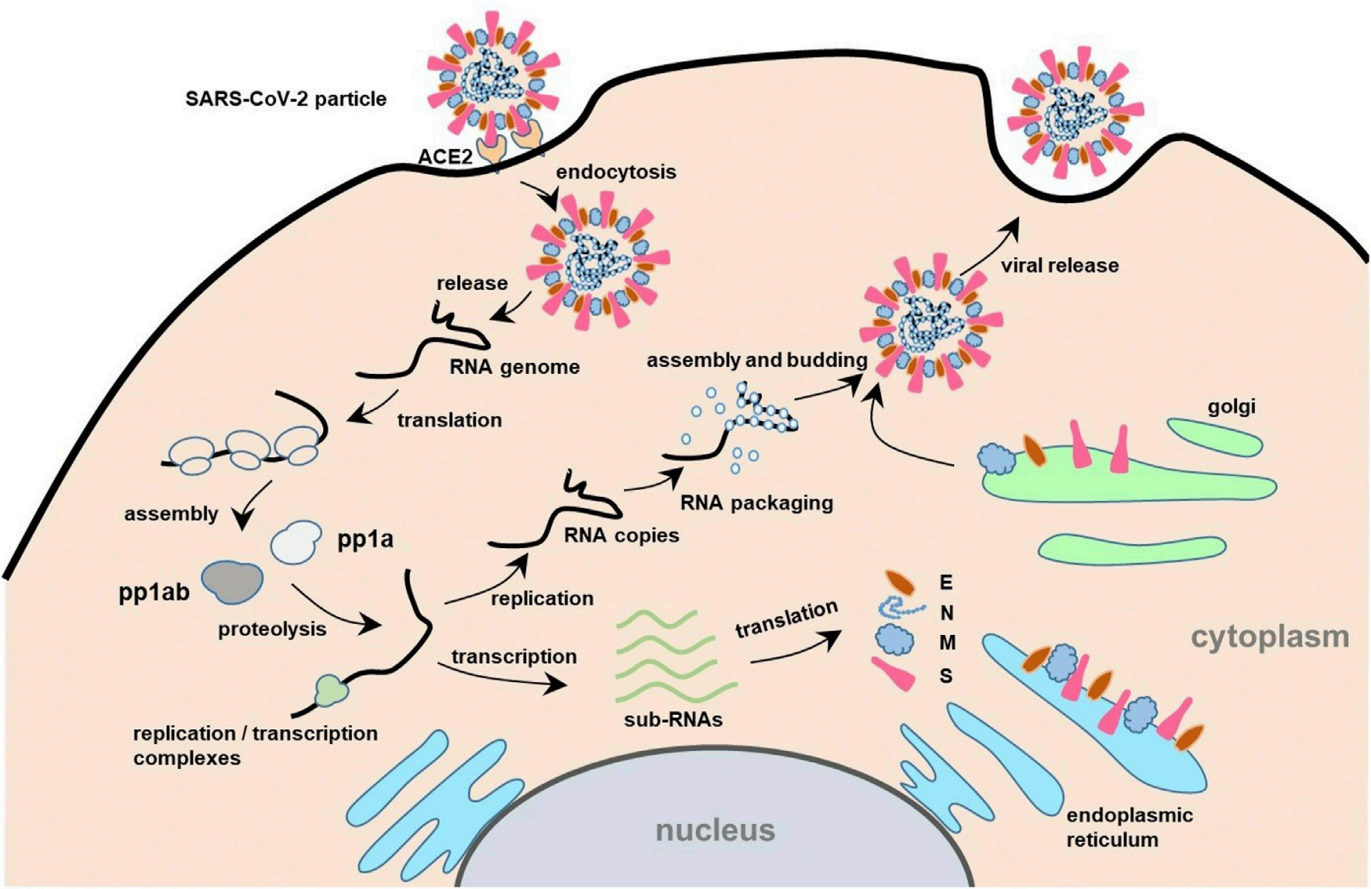
Schematic illustration of the process of SARS-CoV-2 infecting the host and its life cycle. The receptor binding domain (RBD) of S protein of SARS-CoV-2 mediates the infection by binding to angiotensin-converting enzyme 2 (ACE2) receptors on the surface of host cells (Inoue et al., 2007). The virus enters the cell by endocytosis, and then releases the RNA genome. Subsequently, the specific sequence translate polyprotein (pp)1a/1ab are translated to the polyprotein pp1a and pp1ab, respectively. Pp1a and pp1ab are self-cleaved into 16 nonstructural proteins (nsps) by proteolysis (Ji et al., 2020). The nsps coalesce to form replicase/transcriptase complexes containing multiple enzymes. Within the complexes, the offspring RNA is transcribed, which is used as sub-RNA to translate the structural proteins (E, N, M, S) and some auxiliary proteins of the virus (Kim D. et al., 2020). Then, the structural proteins enter the Golgi intermediate region of the endoplasmic reticulum along the secretory pathway to complete self-assembly. Meanwhile, the replicated RNA copies bind to N protein to form a ribonucleoprotein complex. Through the intake of ribonucleoprotein complex by the viral vesicles, the matured viruses are formed and then released outside the cell.

In the first step of infection, the S protein of SARS-CoV-2 binds to the angiotensin-converting enzyme 2 (ACE2) receptors on the surface of susceptible cells and attaches to them (Figure 2) (Kuhn et al., 2004; Letko et al., 2020). The binding of the S protein (*via* the RBD) to the ACE2 receptor triggers the endocytosis of cells and exposes virus particles to cellular proteases. It releases nucleic acids under lysosomal enzymes (Hoffmann et al., 2020). The released viral RNA can be directly attached to the ribosome of host cells to generate early proteins. Meanwhile, ORF1a and ORF1ab encode the replicase polyprotein pp1a and pp1ab respectively, and are involved in the assembly of nonstructural proteins into the virus replication transcription complex (RTC) (Lu et al., 2020; Zhou et al., 2020). With the generation of RTC, the functional RNA is transcribed, translating into the structural proteins and some auxiliary proteins of the virus. The structural proteins then enter the endoplasmic reticulum-Golgi intermediate compartment along the secretion pathway to assemble viral particles (Stertz et al., 2007; Du et al., 2009; Kim D. et al., 2020). Several steps are essential during assembly, including nucleic acid aggregation, capsomere assembly, and nucleic acid filling. Once assembled, the mature virus forms a vesicle, merges with the cell membrane, and is released from the cell by budding to infect other host cells. All these studies provide immense information regarding the SARS-CoV-2 genome and functional proteins, providing the foundation for rapid diagnosis and treatment of the variant strains (Du et al., 2009; Feng et al., 2020).

## Diagnosis Methods

**FIGURE 3:**
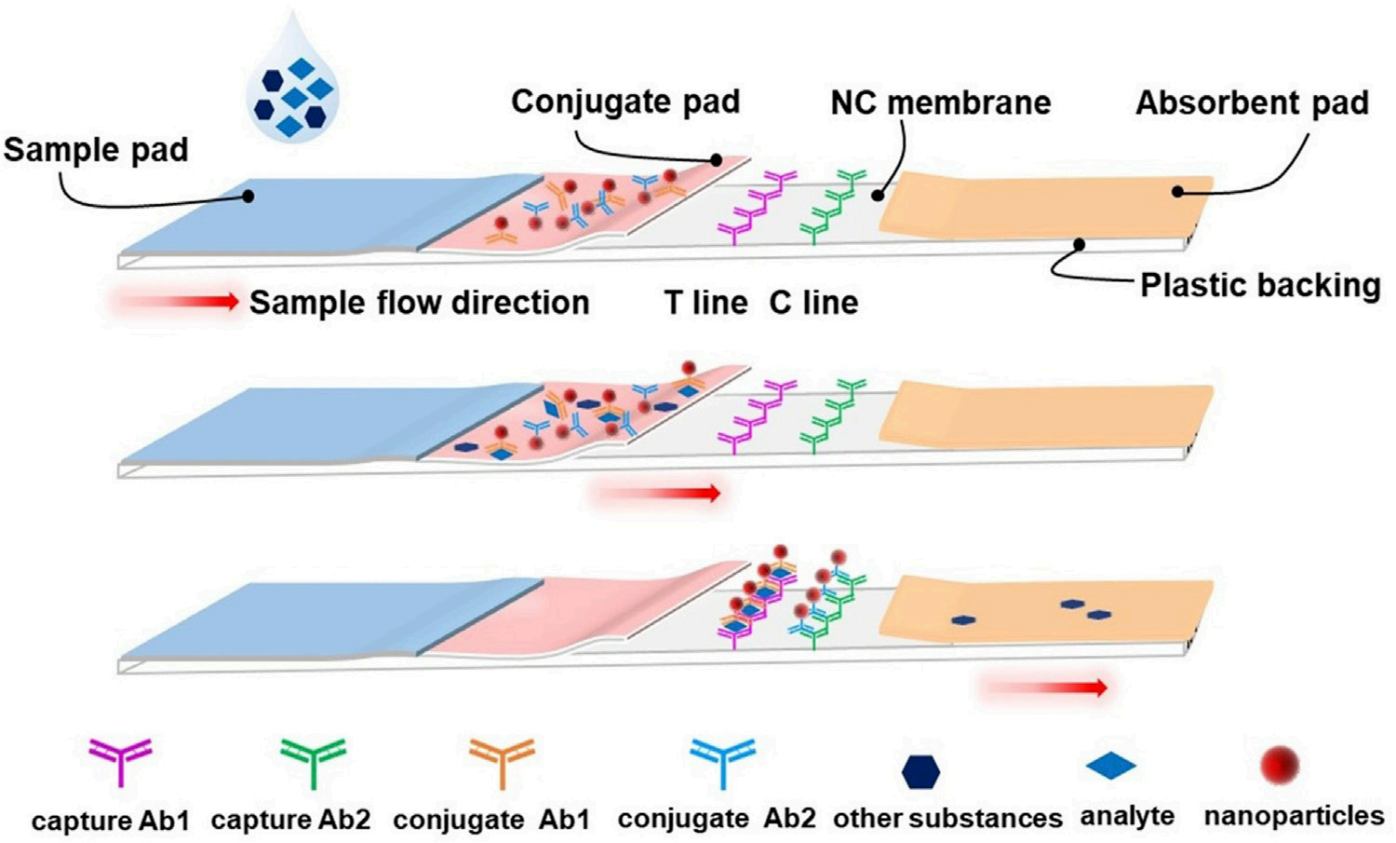
Schematic illustration of a typical lateral flow assay strip. The typical lateral flow strip is composed of a sample pad, conjugate pad, absorbent pad, nitrocellulose membrane, test line (T line), control line (C line) and plastic backing. The nanoparticles (such as AuNPs) ligated with the conjugate antibody are pre-embedded at the conjugate pad, and the capture antibodies are also pre-embedded on the T line and C line, respectively. Upon loading, the liquid sample will flow from the sample pad to the absorbent pad by capillary force. When the analyte passes through the conjugate pad, it will be captured by the corresponding conjugate antibody and form the analyte-conjugate antibody@AuNPs complex. The complexes will be fixed onto the NC membrane by capture antibody on the T line. Meanwhile, another conjugate antibody will be fixed on the C line and serve as a control.

An LFA test strip typically comprises a sample pad, a conjugate pad, a nitrocellulose (NC) membrane, an absorbent pad, a plastic backing, and biological reagents (Figure 3). To perform the assay, the liquid sample is loaded onto the sample pad allowing for it to migrate to the conjugate pad, driven by capillary force. In this process, the target in the sample is captured by a specific antigen or antibody-coated nanoparticle embedded in the conjugate pad. As the complexes formed by the analytes and nanoparticles continue to flow forward, they specifically bind to another antigen or antibody embedded in the NC membrane. After a few minutes, the target is captured and forms a specific signal at the test line (T line) and control line (C line), representing the presence or absence of the target and the successful completion of the reaction. Based on this principle, quantitative determination of analytes can be achieved. One of the most famous applications of the LFA is the home-use pregnancy test strips developed in the early 1970s (Vaitukaitis et al., 1972). Soon after, it was applied in different areas such as food safety (Zhao et al., 2016), agriculture (Wang et al., 2007), healthcare (Choi et al., 2017), forensic science (Old et al., 2009), animal medicine (Shome et al., 2018), and even military (Chao et al., 2017). Currently, it is one of the most helpful tools for controlling the spread of COVID-19.

### Viral RNA-Based LFAs

Among the five ORF regions of the SARS-CoV-2 genome (Figure 1), ORF1ab, N, E, and S regions are usually selected to develop molecular diagnostic tests (Chu et al., 2020; Corman et al., 2020; Sheikhzadeh et al., 2020). The homology of the ORF1ab, N, and S regions with other coronaviruses was low, except in the E region (Sheikhzadeh et al., 2020). E can only be used as a screening region. If only E is positive, it indicates the presence of a coronavirus infection without a specific type (Cui and Zhou, 2020; Zhang W. et al., 2020). SARS-CoV-2 infection can be confirmed through the combined detection of the E and ORF1ab, N or S regions. By changing the detection sensitivity and specificity, the virus can be detected in the early stage of infection. Timely necessary solution strategies are then performed to avoid the occurrence of pandemics and deaths as much as possible. Although qRT-PCR is the main approach for nucleic acid detection, and various kits have been approved by the Food and Drug Administration (FDA), its application has been restricted in some countries and regions that are under developed (FDA, 2020), owing to its high cost that many low-income families cannot afford. Accordingly, there is an urgent need to develop cost-effective methods for early nucleic acid diagnosis for non-hospital and non-laboratory use.

**FIGURE 4:**
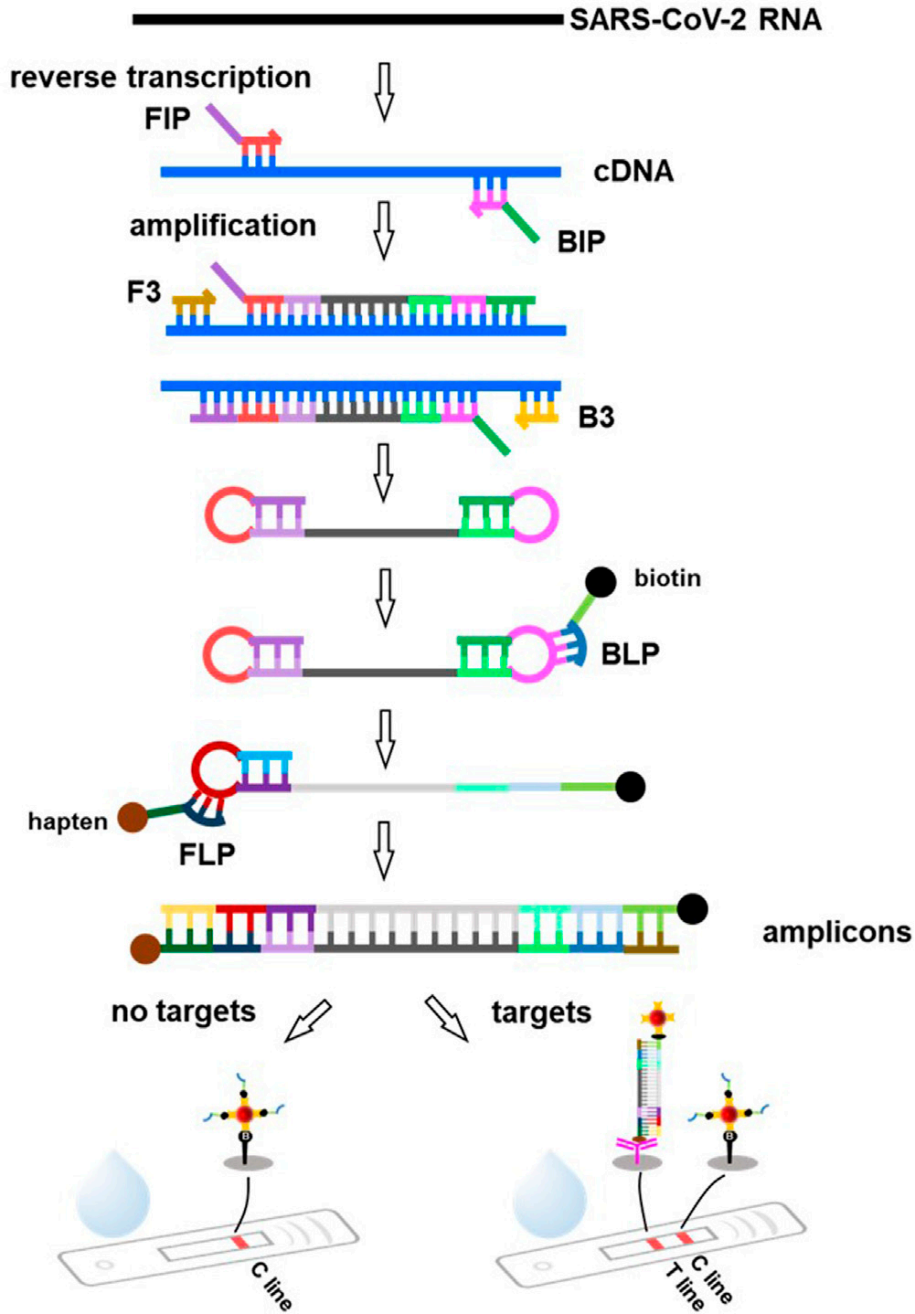
Principle of the lateral flow assay based on RT-LAMP technique. SARS-CoV-2 RNA is reverse transcribed to cDNA, followed by specific amplification with the forward inner primer (FIP), backward inner primer (BIP) and forward primer (F3), backward primer (B3), producing a great many of dumbbell-shaped product. These products are used as templates and amplified by specific labeled primers backward loop primer (BLP), forward loop primer (FLP, such as hapten, biotin) to produce amounts of amplicons. As the amplified products migrate along the FLA strip, the conjugations of amplicons and streptavidin coated AuNPs can be captured by the capture antibody immobilized at the T line, leading to a red visible signal. The excess BLP primers labeled biotin coupled with streptavidin-coated AuNPs keep moving and are captured by biotin immobilized at the C line.

RT-LAMP combined with LFAs is an ideal strategy for developing an inexpensive early diagnostic approach (Figure 4). The RNA (SARS-CoV-2 template) was reverse-transcribed to cDNA as a template for LAMP amplification. Subsequently, the forward inner primer (FIP) initiated isothermal amplification at 65°C, and the new strand derived from the FIP primer was replaced by the forward primer F3 synthesis. Then, the backward inner primer (BIP) and backward primer (B3) anneal to the newly produced strand and extend the sequence to generate a dumbbell-shaped product under DNA displacement polymerase. The product containing the stem-loop can then serve as the template for the second stage of the LAMP reaction. A backward loop primer (BLP) labeled with biotin can anneal to a particular product derived from the LAMP reaction stage. The new products labeled with biotin were also used as a template for the subsequent amplification steps using a forward loop primer (FLP), which was modified with the hapten. As the replication cycle was repeated, many double-labeled detectable products were formed in the mixture (Yan C. et al., 2020; Zhu X. et al., 2020).

Because the amplification product of RT-LAMP cannot be accurately quantified for diagnosis at different stages or viral loads, an LFA can be applied afterwards. As the amplification products migrate along the test strip, biotin-labeled amplification products bind to the streptavidin-coated nanoparticles and are then captured by the specific antibody immobilized on the T line (Figure 4). The excess unreacted BLP- labeled biotin binds to the streptavidin-coated nanoparticles and is captured by biotin immobilized on the C line. If the nanoparticles are colored, the results can be easily visualized with the naked eye. The entire test process can be completed within 30 min (Chen X. et al., 2021). Zhu X. et al. (2020) proposed a method to diagnose COVID-19 by combining multiple reverse transcription loop-mediated isothermal amplification (mRT-LAMP) with nanoparticles-based LFAs, which could simultaneously amplify the ORF1ab and N genes of SARS-CoV-2. The limit of detection (LOD) was 12 copies (for each detection target) per reaction, with no cross-reactions occurring from non-SARS-CoV-2 templates. Similarly, Zhang et al. (2021) reported a one-pot RT-LAMP assay for SARS-CoV-2 based on LFA using clinical samples. The entire contiguous sample-to-answer workflow was completed within 40 min without the assistance of professional instruments and technicians. Importantly, the total accuracy of RT-LAMP for clinical RNA samples was 100%. In another study, a molecular beacon probe was used for the sequence-specific detection of OFR1a amplicons of SARS-CoV-2 LAMP based on the LAMP technique integrated with commercially available LFA strips. The sensitivity of LFA-LAMP was similar to that of qRT-PCR (Varona and Anderson, 2021).

**FIGURE 5:**
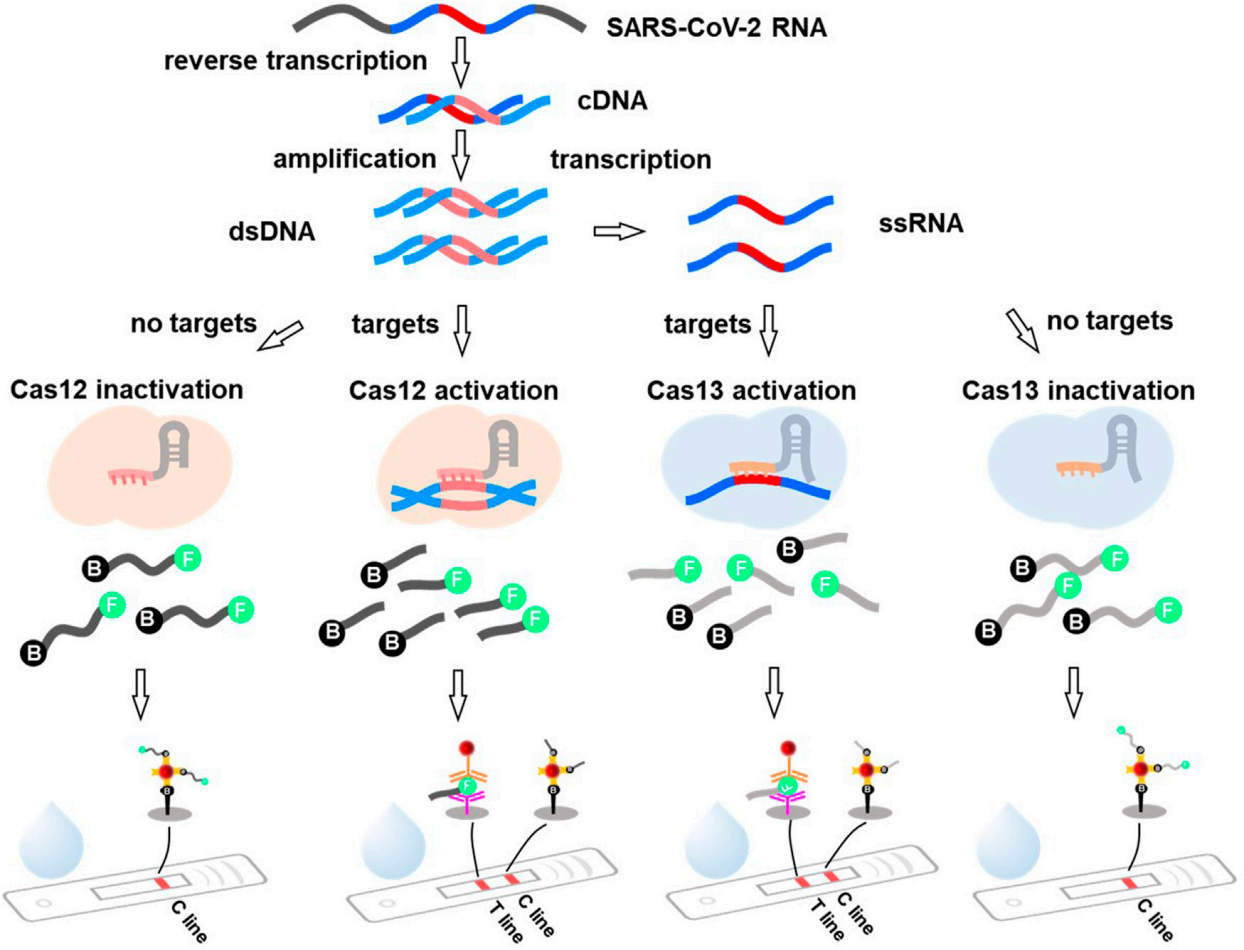
The principle of the CRISPR system mediated lateral flow assay. Viral RNA is first reverse transcribed to cDNA and serve as a template to produce cDNA amplicons. The amplicons are directly added into the CRISPR-Cas12 system and activated the Cas12 by dsDNA with the targeting sequence (the red mark) to cleave the ssDNA (marked with specific recognition molecules at the end, such as biotin and FITC). Alternatively, the amplicons can be transcribed to ssRNA and then added to the CRISPR-Cas13 system. The Cas13 recognizes RNA containing CRISPR targeting sequences and cleaves its ssRNA reporters. The solution containing the cleaved reporters is added to the sample pad. Under the action of capillary tension, part of the reporter-biotin coupled with streptavidin-AuNPs will be captured at C line, and the FITC conjugated antibody-AuNPs @FITC@FITC captured antibody leads to the signal at T line.

Recently, the CRISPR system has been applied to SARS-CoV-2 detection with excellent performance (Flint et al., 2019; He et al., 2020). Therefore, it has been recommended as a potential candidate for LFA-incorporated POCT. CRISPR-LFAs possess the advantages of a CRISPR system with high specificity and sensitivity, as well as convenient and rapid LFAs. Viral RNA is transcribed into cDNA for amplification using isothermal techniques, such as RT-LAMP. The cDNA amplicons are either added directly to the CRISPR-Cas12 system or transcribed to ssRNA under transcriptase and then added to the CRISPR-Cas13 system (Figure 5). This is because Cas12 targets ssDNA whereas Cas13 targets ssRNA (Abudayyeh et al., 2017; Chen et al., 2018). Cas12 is activated by dsDNA with a CRISPR-targeted sequence to cleave ssDNA reporters, and Cas13 recognizes RNA containing the CRISPR-targeted sequence and cleaves its RNA reporters (Figure 5). Reporters, which are short, single-stranded nucleic acids labeled with a fluorophore and quencher at the end, can be used as substrates (Broughton et al., 2020; Gootenberg et al., 2017). Cleavage of the signaling reporter separates the quencher from the fluorophore, releasing fluorescence signals. Subsequently, the released fluorescence signals appear on the T line when the products are captured by the embedded capture antibodies of the LFAs (Figure 5). Furthermore, Patchsung et al. (2020) developed a reverse transcription-recombinase polymerase amplification (RT-RPA)-mediated CRISPR-Cas13a platform called the clinical validation of the specific high-sensitivity enzymatic reporter unlocking (SHERLOCK) assay for the detection of SARS-CoV-2. 42 RNA copies per reaction were detected. Xiong et al. (2021) established a triple-line LFA for the rapid and simultaneous dual-gene detection of SARS-CoV-2 by integrating the CRISPR/Cas9 system with multiplex reverse transcription-recombinase polymerase amplification (RT-RPA). The analysis of 64 nasopharyngeal swab samples showed 100% negative predictive agreement and 97.14% positive predictive agreement. Similar results have been reported by others (Marsic et al., 2021; Osborn et al., 2021; Zhu et al., 2021). Notably, Broughton et al. (2020) proposed a protocol using the DNA endonuclease-targeted CRISPR trans reporter (DETECTR) for SARS-CoV-2 RNA testing. Specifically, isothermal amplification combined with CRISPR/Cas12 DETECTR was used to develop a rapid assay for COVID-19 diagnosis. It provided 95% positive predictive agreement and 100% negative predictive agreement compared to the qRT-PCR assay, possessing huge value for POCT and on-site analysis of SARS-CoV-2 or other viruses in the future.

In addition, a POCT assay based on other nucleic acid hybridization techniques integrated with LFAs has been reported. For instance, the application of the S9.6 monoclonal antibody with high affinity and selectivity to DNA/RNA heterozygotes enabled SARS-CoV-2 detection with high sensitivity and specificity (Wang D. et al., 2020). In another study, through screening more effective reverse transcriptase, optimizing amplification primers, and adding RNase H to improve the amplification efficiency of RT-RPA, the sensitive detection of SARS-CoV-2 RNA was achieved (Qian et al., 2020). Moreover, qRT-PCR combined with LFAs could simultaneously detect RdRp, ORF3a, and N genes of SARS-CoV-2, with a detection limit of 10 copies per test for each gene (Yu et al., 2020). Furthermore, next-generation sequencing (Wu et al., 2021), a microfluidic-integrated RPA (Liu D. et al., 2021), and a catalytic hairpin assembly enzyme-free signal amplification reaction (Zou et al., 2021) coupled with LFAs techniques have been exploited as POCT for rapid diagnosis.

Although qRT-PCR is the primary tool for viral nucleic acid detection, its requirements for a thermal cycler are not ideal for POCT applications. Meanwhile, in recent years, various isothermal hybridization-based LFAs have been developed to determine viral RNA. Taking advantage of this hybridizations and/or amplification techniques, a significant increase in detection signal at a single temperature in a single tube can be achieved without the help of advanced thermal cycle instruments (Wang D. et al., 2020; Zhang et al., 2021). Therefore, it is expected to be developed into a simple, rapid, and widely used analytical method.

### Antibody-Based LFAs

Although viral RNA-based LFAs detection has been exploited for the early diagnosis of COVID-19, it cannot be used to monitor disease progression or identify past infection and recovery. Therefore, antibody testing is important. Antibodies are specific proteins produced by the immune system and can specifically bind to target antigens, mainly determined by the complementary regions at the N-terminus of the antibody. Five types of antibodies have been found in humans: immunoglobulins IgG, IgM, IgA, IgE, and IgD, which are secreted by differentiated terminal B cells (Vitetta et al., 1989; Esser and Radbruch, 1990).

**FIGURE 6:**
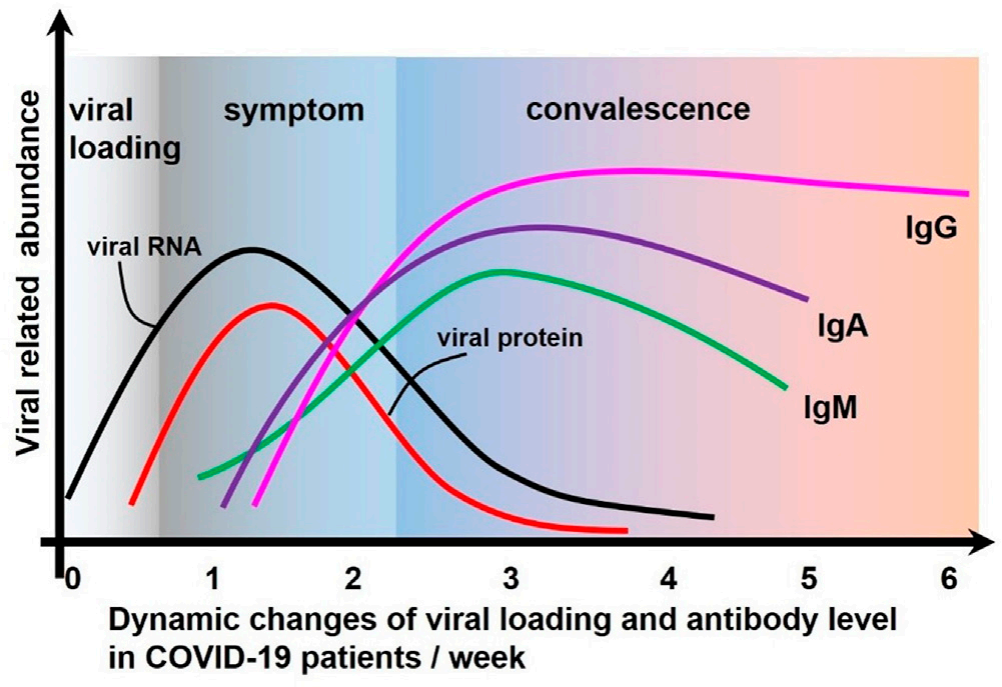
The dynamic process of viral load change and immune response after SARS-CoV-2 attacks the host.

The detection of SARS-CoV-2 usually includes quantitative detection of different types of virus-specific IgG and IgM or the total level of IgG/IgM (Figure 6). At different stages of infection, the state of the host immune system and the characteristics of antibody production are different. According to the accumulated data, IgM can be detected as early as 4 days after SARS-CoV-2 viral infection, peaking around the 20th day, and then declining (Guo et al., 2020). IgG was detected on the seventh day after infection; it then gradually increased, reached a peak between the 21st and 25th day, and then remained at a high level (Theel et al., 2020). IgA increased from 6 to 8 days after infection and peaked between the 18th and 21st day, with a longer duration and higher concentration than IgM but lower specificity (Padoan et al., 2020). Thus, IgM and IgG levels can be used as indicators of early, current, or previous infections. Numerous diagnostic methods for SARS-CoV-2 have been developed through measuring IgM and/or IgG concentrations, especially reaching their peaks between the 20th and 25th day in the serum. LFAs are a powerful means of antibody determination in the POCT setting. Recently, antibody detection based on LFAs for SARS-CoV-2 has been widely reported.

In antibody-based LFAs, fluid samples are loaded onto the sample pad, allowing the sample to flow through an immobilized anti-human antibody band. If anti-SARS-CoV-2 antibodies are present, they can be conjugated with nanoparticles to indicate the signal intensity collected at the T line; if not, the T line is empty, and no signal is detected. Among them, colloidal gold (AuNPs)-based LFAs antibody detection techniques have attracted wide attention because the results can be read directly based on color change, without the aid of instruments. Huang et al. (2020) designed a strategy using AuNPs-based LFAs to achieve the rapid diagnosis and on-site detection of IgM antibodies against SARS-CoV-2. In this study, the SARS-CoV-2 nucleoprotein was coated on an analytical membrane for sample capture, and anti-human IgM was labeled with AuNPs to form the detection reporter. The result coincided with those of RT-PCR by testing the serum samples of COVID-19 patients and healthy individuals. Similar works have been reported by other laboratories (Wen et al., 2020; Cavalera et al., 2021; Elter et al., 2021). Because the results of AuNP-based LFAs are mainly judged visually, the sensitivity is not sufficiently high and will inevitably lead to bias. To improve the sensitivity, a variety of antibodies (such as IgG/IgM) can be measured simultaneously. Alternatively, advanced nanoparticles can also be used. To determine IgG/IgM, Peng et al. (2021) developed the detection of IgG/IgM against the nucleocapsid protein, anti-N IgG/IgM, and RBD in the spike glycoprotein, anti-S-RBD IgG/IgM, of SARS-CoV-2. Any virus targeting IgG/IgM found in the clinical sample was considered positive. The strategy achieved high sensitivity and accuracy. As expected, sensitivity and specificity were 96 and 100%, respectively. A series of similar studies have been reported by other researchers (Li Z. et al., 2020; Peng et al., 2020; Hu X. et al., 2021; Higgins et al., 2021; Hung et al., 2021). For the latter, selenium nanoparticles synthesized by the ascorbic acid reduction of seleninic acid were used to conjugate with nucleoprotein and simultaneously detect anti-SARS-CoV-2 IgM and anti-SARS-CoV-2 IgG on the platform of lateral flow. Based on this technique, the detection limits of IgG and IgM in human serum were approximately 20 ng ml^−1^ and 5 ng ml^−1^, respectively (Wang Z. et al., 2020).

In another study, a lateral flow system based on superparamagnetic nanoparticles (SMNPs) was developed for the simultaneous determination of anti-SARS-CoV-2 IgM and anti-SARS-CoV-2 IgG. In the system, magnetic signal intensity varied with the concentration of IgG/IgM in the clinical sample, with LODs of 10 ng ml^−1^ and 5 ng ml^−1^, respectively (Bayin et al., 2021). Moreover, aggregation-induced emission (AIE) dye-loaded nanoparticles were designed to detect IgM and IgG against SARS-CoV-2 in serum samples at early stage of infection. The auto-fluorescence from the nitrocellulose membrane sample and the excitation background noise were effectively eliminated owing to the emission of AIE dye in the near-infrared (NIR) window. Thus, the signal background ratio was considerably improved, and the signal of IgM and/or IgG could be detected at 1–7 days after symptoms onset, which was earlier than that of the AuNP-based test strip (8–15 days) (Chen L. et al., 2021; Chen R. et al., 2021). Additionally, other composite nanoparticles containing time-resolved fluorescent microspheres (Zhang C. et al., 2020), Ag shells on SiO_2_ core (SiO_2_@Ag) formed SERS nanoparticles (Liu H. et al., 2021), and SiO_2_@Au@QD nanobeads (Wang C. et al., 2020) have been used for the early diagnosis of SARS-CoV-2 based on the principle of LFAs. These assays are summarized in Table 2.

**TABLE 1:** Nucleic acid amplification technique combined with the lateral flow assay for detection of SARS-CoV-2.

Target	Detection technique	Signal readout	LOD	Sensitivity, specificity	Time	Reference
ORF1ab & N genes	CRISPR-Cas12a coupled with LFB	colorimetric	2–10 copies/reaction	100%, 100%	<60 min	Zhu et al. (2021)
ORF8a gene	CRISPR-Cas9 coupled with LFB	fluorescence	—	—	∼60 min	Osborn et al. (2021)
ORF1ab & E genes	CRISPR/Cas9-mediated LFA	colorimetric	4 copies/µl	100%, 97%	<60 min	Xiong et al. (2021)
ORF1ab & N genes	mRT-LAMP coupled with LFB	fluorescence	12 copies/ reaction	100%, 100%	60 min	Zhu et al. (2020c)
E & N genes	RT-LAMP/Cas12-based LFA	fluorescence	10 copies/µl	95%, 100%	30–40 min	Broughton et al. (2020)
ORF1a gene	LAMP integrate with LFIA	fluorescence	2300 copies/ reaction	—	—	Varona and Anderson, (2021)
ORF1ab gene	two sugar barrier modified LFA	colorimetric	0.5 nM	—	—	Tang et al. (2021)
ORF1ab & N genes	a catalytic hairpin assembly (CHA) reaction) coupled with LFIA	fluorescence	2000 copies/ml	—, 100%	<90 min	Zou et al. (2021)
S & N & ORF1ab genes	SHERLOCK lateral-flow readout	colorimetric	42 copies/ reaction	97%, 100%	∼30 min	Patchsung et al. (2020)
N gene	RT-RPA/CRISPR-Cas9 coupled with LFA	colorimetric	2.5 copies/μl	96%, 100%	—	Marsic et al. (2021)
N gene	RT-LAMP-LFA	colorimetric	3.9 × 10^3^ RNA copies/ml	82%	15 min	Agarwal et al. (2022)
N gene	AuNP-LFA	colorimetric	0.02 copies/μl	100%, 100%	<10 min	Dighe et al. (2022)
ORF1ab & N genes	RT-LAMP-LFA	colorimetric	40 copies/μl	100%, 100%	<40 min	Zhang et al. (2021)
RdRp & ORF3a & N genes	lateral flow strip membrane assay	fluorescence	10.0 copies/test	99%, —	30 min	Yu et al. (2020)

**TABLE 2:** Antibody-targeting SARS-CoV-2 detection on the basis of lateral flow assay.

Target	Detection technique	Signal readout	LOD	Sensitivity, specificity	Time	Reference
IgG & IgM	selenium nanoparticle-based LFAs	fluorescence	5 ng/ml, 20 ng/ml	93%, 97%	<10 min	Wang et al. (2020c)
IgG & IgM	colloidal gold-based LFAs	colorimetric	—	—	—	Hu et al. (2021b)
IgG & IgM	time-resolved fluorescence immunoassay with LFAs	colorimetric	0.121 U/L, 0.366 U/L	97%, 99%	15 min	Zhang et al. (2020a)
IgG	hybrid capture fluorescence immunoassay with LFIA	fluorescence	1000 TU/ml	95%, —	<60 min	Wang et al. (2020b)
IgM	AuNP-LF assays	colorimetric	12 copies/ reaction	100%, 93%	15 min	Huang et al. (2020)
IgG	AuNP-LFIA	colorimetric	186 pg/ml		10–15 min	Hung et al. (2021)
IgG	lanthanide-doped nanoparticles-based LFIA	fluorescence	—	—	10 min	Chen et al. (2020c)
IgG	AuNP-LFIA	colorimetric	—	69%, 100%	15–20 min	Wen et al. (2020)
IgG	AuNP-LFA	colorimetric	10^7^ particles/µl	—	5 min	Peng et al. (2020)
IgA	LFIA	Optical /chemilumine scence	—	—	15 min	Roda et al. (2021)
IgG & IgM	LFIA	fluorescence	—	82%, 95%	15 min	Nicol et al. (2020)
IgG & IgM	a giant magnetoresistance based LFIA sensing system	colorimetric	5 ng/ml, 10 ng/ml	—	10 min	Bayin et al. (2021)
IgG & IgM	colorimetric-fluorescent dual-mode LFIA biosensor	fluorescence	1.2 mg/ml, 0.9 mg/ml	100%, 100%	15 min	Wang et al. (2020a)
IgG & IgM	AuNP-LFAs	colorimetric	—	89%, 91%	15 min	Li et al. (2020b)
IgG & IgM	colloidal gold-based lateral flow immunoassay test strips	colorimetric	—	96%, 100%	15 min	Peng et al. (2021)
IgG & IgM	selenium nanoparticle-based LFIA	colorimetric	20 ng/ml, 60 ng/ml	95%, 96%	<10 min	Chen et al. (2022)
IgG & IgM	a commercial POCT LFA	colorimetric	—	88, 93; 93, 100%	—	Hoffman et al. (2021)
IgG & IgM	three different commercial POCT LFIAs (Bioclin, Brazil; Livzon, China; Wondfo, China)	colorimetric	—	86, 100; 48, 100; 44, 100%	—	Chen et al. (2020c)
IgG & IgM	LFA	colorimetric	—	87, 100; 50, 80%	<15 min	Ragnesola et al. (2020)
IgG & IgM	SERS-based LFIA	SERS	1 pg/ml, 1 pg/ml	—	25 min	Liu et al. (2021b)
IgG & IgM	SERS-based LFIA	SERS	1 ng/ml, 0.1 ng/ml	—	15 min	Chen et al. (2021c)
IgG & IgM	SERS-based LFA	SERS	100 fg/ml	—	—	Srivastav et al. (2021)
IgG & IgM	quantum dot (QD) nanotag-integrated LFA	fluorescence	1:10^7^ dilution	97%, 95%	15 min	Wang et al. (2021a)
IgG & IgM	AIE nanoparticle-labeled LFIA	fluorescence	0.125 μg/ ml, 0.236 μg/ ml	95%, 78%	10 min	Chen et al. (2021b)
IgG & IgM & IgA	colloidal gold-coupled LFAs	colorimetric	—	94%, 100%	20 min	Cavalera et al. (2021)
Total antibodies	colorimetric lateral flow immunoassay	colorimetric	—	92%	5–10 min	Ghorbanizamani et al. (2021)

**TABLE 3:** Antigen-based lateral flow assay of SARS-CoV-2.

Target	Detection technique	Signal readout	LOD	Sensitivity, specificity	Time	Reference
S protein	nanozyme and enzymatic chemiluminescence immunoassay with the lateral flow strip	fluorescence	0.1 ng/ml	—	15 min	Liu et al. (2020a)
N protein	fluorescence immunochromatographic lateral flow assay	fluorescence	—	98%, 100%	10 min	Diao et al. (2020)
N protein	fluorescence immunochromatographic lateral flow assay	fluorescence	—	85%, 100%	<15 min	Escrivá et al. (2021)
S & N protein	up-conversion nanoparticles labeled lateral flow immunoassay	fluorescence	1.6 ng/ml, 2.2 ng/ml	—	10 min	Guo et al. (2021)
N protein	half-strip lateral flow assay	fluorescence	0.65 ng/ml	—	20 min	Grant et al. (2020)
N protein	open-access lateral flow assay	colorimetric	2.5 × 10^4^ copies/swab	69, 97; 83, 97%	—	Grant et al. (2021)
recombinant antigen	microfluidic immunoassay system	fluorescence	—	—	<15 min	Lin et al. (2020)
spike RBD protein	LFA based on gold nanospheres	thermal contrast amplification reading	0.016 fg/ml (in buffer), 0.125 fg/ml (in nasopharyngeal wash)	—	30 min	Liu et al. (2021c)
N protein	phage display technology integrated with LFIA-based biosensor	colorimetric	2 ng	—	20 min	Kim et al. (2021)
antigen cocktail	colorimetric lateral flow immunoassay	colorimetric	—	93%	5–10 min	Ghorbanizamani et al. (2021)

Generally, antibody-based LFAs are sensitive and accurate. It can also evaluate the stages of SARS-CoV-2 infection according to the level of IgG and/or IgM in the test samples to provide a theoretical reference for early quarantine, treatment, and prognosis recovery. For infection monitoring and epidemiology studies, it can be a crucial tool in the post-pandemic era.

### Antigen-Based LFAs

The purpose of antigen detection is to directly determine the viral proteins of SARS-CoV-2, such as N or S proteins. When SARS-CoV-2 attacks the host, its structural proteins increase along with the proliferation of the virus. At the early stage, the corresponding antibodies produced by the immune system are not sufficiently high to be detected. If a virus can be directly detected earlier, more time could be left for subsequent quarantine and treatment. Therefore, there is an urgent need to develop an approach to determine antigens with high sensitivity and specificity. The LFA-based method has unique advantages in antigen detection, such as convenient, speed, and suitability for various application scenarios.

The principle of LFAs antigen detection is based on the double-antibody sandwich method. Therefore, it is essential to screen for an antibody pair, named capture antibody and detection antibody, for the antigen capture by SARS-CoV-2. It often requires tremendous labor to screen for the correct antibody pairs. The selected antibody needs to be specific to avoid cross-reaction with severe acute respiratory syndrome coronavirus (SARS-CoV) or middle east respiratory syndrome coronavirus (MERS-CoV) because the essential functional domains of SARS-CoV-2 are highly homologous (Park et al., 2020). Kim et al. (2021) used phage display technology (Ledsgaard et al., 2018) to screen single-chain variable fragment (scFv)-crystallizable fragment (Fc) fusion proteins as specific antibodies for the detection of SARS-CoV-2 N protein.

In this study, specific clones for the SARS-CoV-2 N protein were primarily selected by three rounds of biopanning and ELISA in the phage library, which contained many phages displaying different scFvs. scFv-Fc fusion proteins were then generated based on the above sequences and confirmed by confirmatory ELISA. Newly developed scFv-Fc antibodies are specifically bound to the antigen of SARS-CoV-2. When the sample is loaded onto the platform, the virus protein is captured by detection antibodies, and a double-antibody sandwich complex (capture antibody-antigen-detection antibody) is formed in the test region. The S protein can be a candidate for antigen selection because there is no cross-reaction with other coronaviruses such as MERS-CoV and SARS-CoV (Yan R. H. et al., 2020). Moreover, Liu D. et al. (2020) screened antibody pairs for S-RBD antigens using ELISA and nanoenzyme colorimetric strip approaches. The assay for the antigen S protein of SARS-CoV-2 based on the LFAs platform was then performed. This method demonstrated high sensitivity and specificity and the results could be detected using a simple signal reader. Similar antigen detection approaches for SARS-CoV-2 have been reported in other literatures (Grant et al., 2020; Lin et al., 2020; Guo et al., 2021).

Generally, in the early stage of SARS-CoV-2 infection, when the nucleic acid test is negative and the antibody level is undetectable, antigen detection has a specific value. A series of experiments were performed to evaluate the sensitivity and specificity of the SARS-CoV-2 antigen diagnostic test. The results showed that when the viral load was high in the first week, the sensitivity and specificity were more than 90 and 100%, respectively (Porte et al., 2020). As more LFA-based antigen detection kits are approved, they will be more widely used in pandemic prevention, especially in public places such as homes and airports.

### Other Emerging LFAs

Apart from detecting viral RNAs, the corresponding antibodies and the surface antigens, LFAs-based detection of a whole virus particle or other new biomarkers may be an interesting detection strategy in the field of rapid detection. Deng J. Q. et al. (2021) designed a method using an aptamer and polyethylene glycol (PEG) to directly detect viral particles of SARS-CoV-2. The assay relied on the high-affinity binding to the S protein by the screened aptamer and rapid accumulation of viral particles towards the laser spot through PEG-enhanced thermophoresis. The advantages of high-affinity aptamer include their good specificity, high sensitivity, and rapidity. Thus, aptamers can be widely used for the detection of SARS-CoV-2. Similar studies have been reported by others (Chen et al., 2020b; Zhang L. et al., 2020; Song et al., 2020; Acquah et al., 2021; Devi and Chaitanya, 2021; Sun et al., 2021). Although aptamers have not been combined with LFAs for direct diagnosis of virus particles so far, it is believed that the assay, combined with the merits of aptamers and LFA is a promising tool for rapid detection of SARS-CoV-2 in the future.

Besides, it has been reported that there exists an abnormal microRNA expression in patients with COVID-19 (Kim W. R. et al., 2020; Sacar Demirci and Adan, 2020; Donyavi et al., 2021; Marchi et al., 2021). An abnormal expression of microRNA is closely related with the occurrence and development of various diseases and can be used as a potential biomarker for specific conditions. Therefore, it is feasible to develop a method based on LFAs to simultaneously detect a variety of differentially expressed microRNAs related to SARS-CoV-2 to achieve an indirect diagnosis of the virus of COVID-19 infection rapidly. In addition, other biomarkers such as D-dimer, CD^4+^, CRP protein, or cytokines can be used to exploit some rapid detection methods for SARS-CoV-2 in the POCT setting.

Furthermore, with the continuous emergence of new strains of SARS-CoV-2, their infectivities and pathogenicities are different (Volz et al., 2021; Zhang et al., 2022). The corresponding aptamers can be screened according to the characteristics of the variant strain, including the shape of the virus particles, characteristics of the RNA conserved region, and structure of functional proteins. Combined with LFA, methods that can rapidly distinguish between various mutated viruses have been developed. Moreover, the severity of symptoms caused by variant viruses may lead to differential levels of these biomarkers. Thus, a novel method can be developed based on LFA to detect different subviruses by comparing the differences in these biomarkers.

## Conclusion and Future Perspectives

Compared to the SARS-CoV-1 outbreak in 2003, SARS-CoV-2 has caused unprecedented damage to society. Over the past 2 years, progress has been achieved in all aspects to control the dissemination of the virus, especially in terms of establishing a medical record system, improvements in medication, vaccine development, and developing diagnostic test kits. Based on the data collected thus far, diagnostic kits mainly entail detecting the RNA of SARS-CoV-2 with the assistance of PCR techniques and viral proteins *via* ELISA assays. Most of these approaches are expensive and/or require operation in a central laboratory or with the help of complicated equipment. Their application is limited, especially in developing countries and regions where the laboratory infrastructure is weak. LFA-based diagnostic assays possess significant merits such as low costs, rapid testing, and easy deployment in any setting. Herein, we focus on the structure and infection process of SARS-CoV-2 and summarize novel LFA-based methods for detecting the virus. The principles and properties of these methods are described in detail to facilitate better understanding and comparison. LFA-based virus tests are suitable for various detection scenarios, including homes, schools, factories and shopping malls. This shows considerable potential for the mass screening of the SARS-CoV-2.

Although numerous LFA-based COVID-19 detection methods have been developed in a short time, few POCT diagnostic kits has been approved by the FDA. A great challenge lies in the robustness and effectiveness of these methods. For example, deformed nanoparticles affect the flow uniformity, the properties of the dye loaded in nanoparticles will deteriorate, and the antibody embedded in the conjugate pad or cellulose membrane will be denatured. There are also non-specific adsorption and steric hindrance effects. All of these factors may affect the stability and reproducibility of the results. Additionally, various other methods have some drawbacks. For viral RNA-based LFAs diagnosis, sample pretreatment involves shipping and storage of specimens, extraction, and purification of RNA, which are critical for the accuracy of diagnosis. Moreover, with the emergence of novel variant strains (Deng X. et al., 2021; Wang et al., 2021c; Davies et al., 2021; Wang et al., 2021d; Lu et al., 2021; Rao Us et al., 2021), the development of effective LFA kits for the detection of mutated strains is challenging. Although the availability of updated genomic data enables the adoption of these assays for mutants, the assays cannot provide information for patients who have already recovered from COVID-19. Immunological assays can avoid this issue because of the relatively conserved structure and amino acid sequence of the viral protein. However, it also faces other problems, such as the specificity, sensitivity, and effectiveness of antibody pairs. Efforts should be made to further improve the performance of these assays and make them more applicable for the early detection of COVID-19.

The following strategies should be implemented for all these challenges. More durable materials and reagents are essential to improve the performance of LFAs system. A standard workflow of LFAs diagnostic assays needs to be established. In addition, it is necessary to conduct a multi-prong diagnosis to confirm the test results and reduce the percentage of false positives and false negatives. For instance, one strip can simultaneously detect the RNA, virus antigen, and the corresponding antibody. In the same case, more than two methods can be simultaneously used at the same time. The complementary advantages of these methods can significantly improve detection accuracy. Furthermore, establishing a monitoring network is of great importance in controlling the spread of COVID-19. For example, smartphone-based automated reader technology has been used to test drug dosage in patients (Carrio et al., 2015). In this regard, a smartphone-based LFA can be used to monitor COVID-19 on time by uploading data to the network. The internet of medical things (IoMT) of a 5G-enabled fluorescence sensor can also be adopted to determinate and monitor COVID-19 online. Various artificial intelligence algorithms, including IoT, big data, and cloud computing, can make collective efforts to achieve a rapid and accurate diagnosis and control of COVID-19. This convenient and fast diagnosis will become a regular practice in the post-pandemic era, whether for SARS-CoV-2 or other viruses.
